# Increased IgE Deposition in Appendicular Tissue Specimens Is Compatible with a Type I Hypersensitivity Reaction in Acute Appendicitis

**DOI:** 10.1155/2021/4194859

**Published:** 2021-10-18

**Authors:** Nuno Carvalho, André Barros, Hélder Coelho, Ana Cóias, Pedro Botelho, Brigitta Cismasiu, Luís Moita, Paulo Costa

**Affiliations:** ^1^Serviço Cirurgia Geral, Hospital Garcia de Orta, Almada, Portugal; ^2^Faculdade de Medicina, Universidade de Lisboa, Lisboa, Portugal; ^3^Innate Immunity and Inflammation Laboratory, Instituto Gulbenkian de Ciência, Oeiras, Portugal; ^4^Serviço de Patologia, Hospital Garcia de Orta, Almada, Portugal; ^5^Instituto de Histologia e Biologia do Desenvolvimento, Faculdade de Medicina, Universidade de Lisboa, Lisboa, Portugal

## Abstract

**Background:**

IgE mediates type I hypersensitivity reaction and can be found in the mucosa of organs affected by allergy. Acute appendicitis (AA) is a common disease, but its etiology remains poorly understood. Here, we investigated IgE deposition in histological sections of AA samples to test the hypothesis that an allergic reaction may substantially contribute to the pathophysiology of AA.

**Materials and Methods:**

In a retrospective study, we assessed the presence of IgE in appendicular specimens of histologically confirmed appendicitis and in the control group, comprised of negative appendicitis and incidental appendectomies, using a monoclonal antibody against human IgE. Samples from 134 appendectomies were included: 38 phlegmonous and 27 gangrenous appendicitis from the study group and 52 incidental appendectomies and 17 negative appendicitis from the control group. The slides were visualized by light microscopy, and a standard procedure was used to manually count the positive IgE staining cells.

**Results:**

IgE staining was present in the cells of all but 5 appendicular specimens. We found a significantly increased number of IgE-positive cells in phlegmonous AA (median = 28) when compared to incidental appendectomy (median = 17) (*p* = 0.005; *p* < 0.0001 when adjusted for age and gender). No difference was found for gangrenous appendicitis. *Discussion*. The presence of IgE supports the contribution of an allergic reaction for the pathophysiology of phlegmonous appendicitis. The reduced number of IgE staining cells in gangrenous appendicitis can be due to tissue destruction, or, as been claimed by others, gangrenous appendicitis is a distinct entity, with different etiology.

**Conclusion:**

In this study, phlegmonous appendicitis had the highest number of IgE-positive appendicular cells. These findings suggest that an allergic reaction can contribute to the pathophysiology of AA, opening a novel possibility for preventive measures in a disease that typically requires surgery.

## 1. Introduction

Acute appendicitis (AA) is the most frequent abdominal surgical emergency. Its etiology remains poorly understood, but it is assumed that endoluminal obstruction is the primary event [1]. However, in clinical practice, obstruction has been rarely observed [1]. Other causes should therefore be considered. Aravindan proposed that an allergic reaction could be one such cause [2] based on the observation that the histological features of AA are similar to those found in allergic reactions, including muscular edema, eosinophilic infiltrate, and mastocyte degranulation [2].

Type I hypersensitivity reaction, or allergy, is the clinical manifestation of an immune response against allergens, which are inducers of immunoglobulin E (IgE) synthesis [3]. Every day, thousands of antigens pass through the gastrointestinal tract of an adult person where they face the intestinal immune system, but an allergic reaction may only occur against a very small number [4]. IgE is synthesized in the affected organ [5]. The final effector of the allergic reaction is the mastocyte degranulation with release of inflammatory mediators, when IgE antigen specific binds to high-affinity receptors [3, 6].

Using a monoclonal antibody against human IgE, we have measured the presence of IgE in appendicular specimens of patients with clinical diagnosis of AA and in incidental appendectomies. If AA is the result of an allergic reaction, it is expected that IgE tissue deposition is higher in AA.

## 2. Objective

The objective of this paper is to measure the presence of IgE in the specimens of patients with clinical diagnosis of AA and in incidental appendectomies.

## 3. Material and Methods

### 3.1. Patients and Study Design

The study was performed in a 600-bed tertiary public hospital, which provides medical care to a population of 280,000 urban habitants. Both surgical and pathology departments are Associate Academic Centres of Faculdade de Medicina da Universidade de Lisboa.

During the study period, all of the patients with the clinical diagnosis of AA were included as potential candidates. The only exclusion criteria were patients aged under 18 years old that were in the paediatrics department care.

Two groups of patients were studied for the presence of IgE in appendicular specimens. The study group consisted of patients that presented to the emergency department with abdominal right lower pain between April 2016 and June 2017 and had histologically confirmed AA. The control group included patients that presented to our department in the same time period with clinical diagnosis of AA, were submitted to appendectomy, but had normal histological findings (NHF), classified as negative appendicitis, as well as patients that were submitted to incidental appendectomy (IA) (International Classification of Diseases ICD-9, code: 4719) defined as the removal of a normal appendix along with the treatment of another pathology, between January 2010 and June 2017.

### 3.2. Ethical Considerations

The study protocol was approved by the Ethics Committee of Hospital Garcia de Orta (Reference 7/2016) and conducted in accordance with the Helsinki Declaration.

### 3.3. Pathologic Analysis

Acute phlegmonous (suppurative) appendicitis (APA) was defined as neutrophilic infiltration in the *muscularis propria*, and acute gangrenous appendicitis (AGA) was defined as necrosis of appendicular wall in a background of transmural inflammation [1]. Appendicular abscess and perforation are often associated with gangrene; therefore, these cases were classified as AGA [8].

The presence of neutrophils in the mucosa was considered a normal variant without clinical significance [9]. The specimens were classified as negative AA when no neutrophil infiltrate was shown in the *muscularis propria* [10, 11].

All the surgical specimens have been proceeded according to our department protocols. The original histology reports were not used. A dedicated GI pathologist reviewed all cases without having previous information from the clinical data or the pathological reports (HC). The evaluation of IgE-positive cells is not part of the pathology lab routine, so this was exploratory. Usually, there are no specific fields for evaluation, as during the most common diagnosis there is no special procedure for the microscopic evaluation of any specimens. Nevertheless, when a new analysis was needed, we would cut new paraffin sections and perform the selected method.

IgE detection was performed by immunocytochemistry (IHC) with the “Optiview DAB IHC Detection Kit,” Ventana® (Roche), detection system on a Ventana® BenchMark ULTRA, according to the manufacturer's protocol. Formalin-fixed, paraffin-embedded appendix sections (3 *μ*m) were stained. The primary antibody was 1/400 dilution (24 min, 36°C) rabbit anti-IgE (RP022), Diagnostic Biosystems, antigen recovery buffer (CC1 32 min, 97°C). The same appendicular specimen was used as external positive control in all slides.

**Figure 1:**
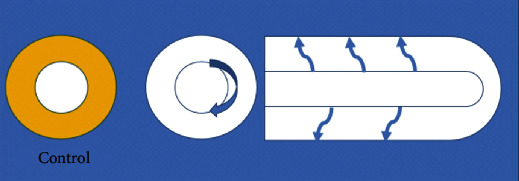
Light microscopy evaluation.

The slides were visualized by light microscopy (“Nikon® ECLIPSE Є400”). We counted the positive IgE cells with bright-brown membrane or cytoplasm (mastocytes or plasma cells, respectively). Diagram 1 tried to represent the aleatory, but reproducible, way; we evaluated all specimens to avoid counting errors and to reproduce the same method in all slides of all section's specimens. First, we scanned at a low magnification and then at high magnification, which was at ten high power fields (objective 40x, 22 mm diameter) (Figure 1). We then counted positive staining cells for IgE and moved the fields clockwise to guarantee that we would not count twice the same field. We evaluated the mucosal and submucosal circularly, and we did the radial evaluation afterwards. All cases had representative circular sections and the apex from the appendix.

### 3.4. Statistical Analysis

For continuous variables, an assessment of the data distribution was performed using histograms, as well as using machine learning techniques, such as random forests, to determine the best distribution family for downstream regression analyses. This type of analysis not only is robust and flexible but also can account for confounding factors [12]. For categorical, binary variables, such as gender, a logistic regression was performed. In both cases, post hoc analyses were performed using estimated marginal means, with Tukey's correction for pairwise comparisons [13].

Statistical analysis was performed on R (https://cran.r-project.org), using ggplot2 [7] and performance [14] packages for determining the data distribution, stats and MASS [15] for regression analysis, and emmeans [16] for estimated marginal means.

### 3.5. Allergy Status

The presence of an allergy was determined by the medical records of the patient, namely the anesthesiologist registration operative files, due to the retrospective nature of the study. The type and prevalence of allergy were presented as follows: 15% of incidental appendectomy corresponded to 8 cases of allergy: 3 related to acetylsalicylic acid, 2 to penicillin, 1 to pyrazalone, 1 to codeine, and 1 to acetaminophen. For NHF, 17% of cases corresponded to 2 cases of allergy to penicillin and 1 to ceftazidime. The prevalence of allergy for AA was of 15%. For APA, there were a total of 6 cases: 3 cases of allergic rhinitis, 2 cases of allergy to penicillin, and 1 to metibasol. For AGA, there were a total of 4 cases: 1 case of asthma, 1 case of allergic rhinitis, 1 case of allergy to penicillin, and 1 to cefuroxime. No type of allergy test was performed.

### 3.6. Other Data

The present study is part of a research project where some collected data had not yet been evaluated. Absence of peritonitis, localized or generalized peritonitis, open or laparoscopic appendectomy, complications, length of hospital stay, and other histologic features were also evaluated but not herein presented, such as the appendicular wall eosinophil infiltration, or some particular characteristics of the appendicular specimens, such as follicular hyperplasia (some data is not shown).

## 4. Results

### 4.1. Patient Group Characteristics

The study population included 134 cases (Table 1).

There was 52 IA, including 20 patients in the context of bladder cancer, 18 in the context of ovarian cancer, and 14 miscellaneous causes.

Based on a regression analysis with a normal distribution, an age difference between patients with IA and the remaining groups was uncovered, with this patient being older than the remaining types of appendicitis (*p* < 0.001). In regard to gender, there were no statistically significant differences among groups. A personal history of allergy was present in approximately 15-17% of each group.

### 4.2. IgE Determination

**Figure 2:**
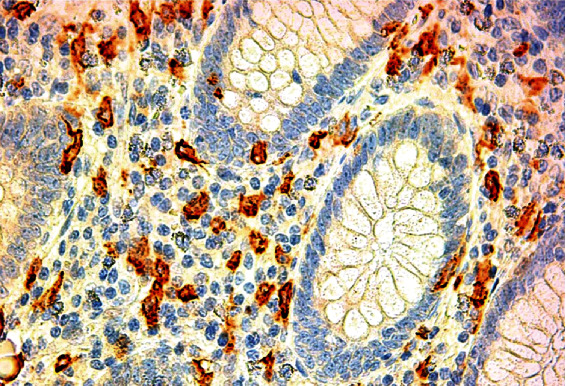
IgE immunostaining, acute appendicitis.

**Figure 3:**
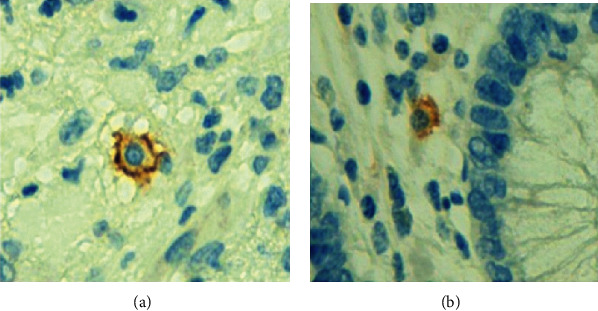
(a) IgE immunostaining, mastocyte. Cytoplasm membrane. (b) IgE immunostaining, plasmocyte. Cytoplasm.

IgE antibody signal (Figure 2) was present at the membrane (Figure 3(a)) and intracytoplasmic levels (Figure 3(b)).

IgE was found in normal appendicular mucosa, namely in 48 out of 52 cases of incidental appendectomy. Clearly, the number of IgE antibody staining cells was reduced from the mucosa to serosa. The distribution of IgE in different histological types is presented (Table 2).

When looking at the distribution of the IgE variable (figure S1), a clear negative skew (high frequency of low levels) and a very low frequency of high values can be observed. After validating this conclusion with the machine learning algorithm in R's package performance [14], we decided to perform a glm using the negative binomial family.

**Figure 4:**
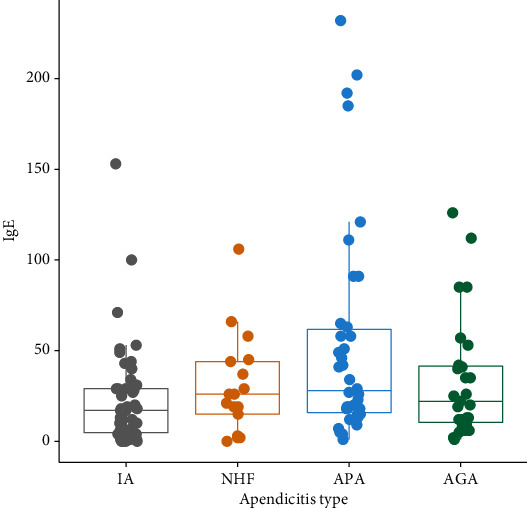
IgE levels according with appendicitis type.

There were important differences among the groups (*p* = 0.005 for the univariate model; *p* = 0.002 for the multivariate model). The distribution of IgE was higher in the APA group than that in the other groups with an IgE median (Q1-Q3) level of 28.0 (15.8–61.8). In pairwise comparisons (Figure 4), the only statistically significant result was for the pair APA versus IA (*p* = 0.0005 for the univariate model; *p* < 0.0001 for the multivariate model).

There were no differences between the other groups: IA versus NHF, *p* = 0.7266; APA versus NHF, *p* = 0.2409; AGA versus NHF, *p* = 0.9894; IA versus AGA, *p* = 0.3610; and APA versus AGA, *p* = 0.2799. The difference between IA and APA is still maintained when we perform a multiple regression analysis to correct for age and gender (*p* < 0.0001) while, apart from APA-AGA (*p* = 0.0314) and IA-NHF (*p* = 0.0409), the others remain nonsignificant. This regression outperforming the univariate one using only appendicitis type (AIC multiple = 1209.311 vs. AIC univariate = 1214.901), suggesting age and gender should be taken into consideration.

## 5. Discussion

There is still considerable discussion about the nature of the etiology of AA [1]. Histological features of type I hypersensitivity reaction have been found in AA leading to the suggestion of an allergy as a possible etiology [2]. The appendicular mucosa contains all the cellular types, B cells, T cells, and Langerhans cells that are required for an IgE immune response [17]. IgE mediates type 1 hypersensitivity reaction, with systemic and local anaphylaxis, sensitizing mast cells and basophils releasing mediators that cause spasm and edema and compromise mucosa blood flow [6, 18]. This reaction can happen in all segments of the intestine but the appendix, with its reduced lumen and a limited capacity to accumulate edema, which is more prone to mucosa ischemia [19]. In allergy, antibodies are synthesized in the target organ, and their presence in the blood is the consequence of an excessive local production [5]. For instance, IgE is found at the nasal mucosa of patients with allergic sinusitis [17, 20].

If AA is mediated by an allergic reaction, it would be expected that the IgE levels on the appendicular tissue, the target organ, are higher in AA than that in the control group, IA, and NHF. A significant difference was found between the 4 groups, APA, AGA, NHF, and IA. In the pairwise analysis, the difference was maintained between APA and IA. These results are compatible with an allergic reaction hypothesis for AA. In fact, in IA, no clinical and histologic features of appendicitis are present, and the number of IgE-positive cells in appendicular tissue is lower than that in the APA group.

No difference was found between APA and NHF. It is well known that a substantial proportion of histologically normal appendices had clear evidence of an inflammatory molecular response in the form of increased cytokine expression, including TNF alpha and IL-2 mRNA [21]. Also, when faced with a macroscopic normal appendix, only a few sections are examined, which are often found to be normal. However, if serial sections are evaluated, focal inflammation can be found [22].

It is possible that patients with pain in the right iliac fossa and no histologic criteria of AA are at an initial phase of AA, and as the neutrophil infiltrate has not yet occurred, the histological diagnosis of AA cannot be made.

No difference was found between AGA and NHF or IA. Tissue destruction in AGA is so extensive that IgE can also be destroyed, and so, no anti-IgE fixation can be documented [22, 23]. Increasing volume of data shows that necrosis of the appendix is a disease different from simple inflammation [24]. A positive association of Th1 response and gangrenous appendicitis was shown [24]. AGA etiology may be different from APA, without the allergic component, and therefore, IgE fixation is not present.

Appendicular abscess and appendicular perforation are clinical diagnoses, having been classified as AGA. In fact, it is well known that intraoperative assessment of the appendix frequently does not concur with histologic findings, and so, the appendicitis classification was based on histology, a more objective criterion [25, 26].

IgE-positive nasal cells in allergic rhinitis consist mainly of mast cells. In our study, no specific coloration was used for evaluating different cell types; it was assumed that most of the cells that fixed anti-IgE at membrane were mastocytes and at cytoplasm were plasma cells (Figures 2, 3(a), and 3(b)). An experimental model of AA in rabbits and specimens of AA in humans showed *lamina propria* infiltration by IgA, IgM, and IgG, but IgE was not studied [22, 27]. Nearly two-thirds of viable mononuclear cells from surgically removed appendices were B cells, composed of sIgM, sIgA, and sIgG [28]. Again, no reference was made to IgE.

To the best of our knowledge, this is the first study assessing IgE in appendicular specimens. The difference of IgE levels in APA and IA are in accordance with a hypersensitivity local reaction responsible for local inflammation in appendices. At a time when antibiotics can prevent some patients from undergoing appendectomies [29], our results offer the possibility of an exciting novel approach to AA management and prevention by desensitization to identified allergens avoiding invasive surgical procedures whenever possible.

Recent evidences suggest the presence of an allergic reaction in AA [30–32]. A clinical study showed that Th2 cytokines, IL-4, IL-5, and IL-9, which are known to be involved in allergic responses, are elevated in phlegmonous AA lavage fluids compared with appendectomy specimens obtained after clinical suspicion of AA not histologically confirmed [30].

A cohort study of 605 children undergoing appendectomy showed that those with IgE-mediated allergy had a 3-fold lower risk of complicated appendicitis compared with those without allergy [31].

A case-control study using a national sample cohort showed an increased risk of appendectomy in asthma patients [32].

In our study, the prevalence of allergy was evaluated according to patient clinical history and medical records, although no difference was observed between groups. At least 20% of the Swedish population suffers from one or several of the clinical manifestations of atopic allergy [33]. Unfortunately, there is currently no data available for the general prevalence of allergy in the Portuguese population.

### 5.1. Strengths

We have straight and uniform definitions of APA, AGA, NHF, and IA. Using a methodology never applied in appendicular mucosa, anti-IgE antibody staining, we herein show that a mediated IgE reaction is present in inflammatory appendicular tissue, and so, AA could be the result of an allergic reaction.

### 5.2. Limitations

Study performed in a single center, with a reduced number of cases. These results should be confirmed in larger cohorts and by others.

## 6. Conclusion

In this study, the higher number of IgE-positive cells in appendicular specimens was found in acute phlegmonous appendicitis, suggesting that an allergic reaction can be involved in the pathophysiology of acute appendicitis. A novel opportunity for preventive measures in a disease that typically requires surgical intervention is introduced.

## Figures and Tables

**Table 1:** Appendicular specimen's demographics.

	IA (*n* = 52)	NHF (*n* = 17)	APA (*n* = 38)	AGA (*n* = 27)	*p* value
Age (y)	64 (55–70)	32 (27–40)	37 (25–48)	38 (26–48)	<0.001 (LR)
Sex (M/F)	29 (55.8)/23 (44.2) (*n* = 52)	3 (17.6)/14 (82.4) (*n* = 17)	14 (36.8)/24 (63.2) (*n* = 38)	13 (48.1)/14 (51.9) (*n* = 27)	0.034 (LgR)
Allergy	8 (15.7) (*n* = 51)	3 (17.6) (*n* = 17)	6 (16.7) (*n* = 36)	4 (14.8) (*n* = 27)	0.095 (LR)

IA: incidental appendectomy; NHF: normal histologic findings; APA: acute phlegmonous appendicitis; AGA: acute gangrenous appendicitis. Results presented as median (Q1-Q3) for continuous nonparametric variables or number (valid percentage) of subjects for categorical variables. LR: indicating the corresponding *p* value was obtained using a linear regression. LgR: indicating the corresponding *p* value was obtained using a logistic regression.

**Table 2:** The distribution of IgE in appendicular specimens.

	*N* (%)	Median (Q1-Q3)	Min	Max
IA	52 (39)	17.0 (4.8-29.0)	0	153
NHF	17 (13)	26 (15-44)	0	106
APA	38 (28)	28 (15.8-61.8)	1	232
AGA	27 (20)	22 (10.5-42)	1	126

IA: incidental appendectomy; NHF: normal histologic findings; APA: acute phlegmonous appendicitis; AGA: acute gangrenous appendicitis.

## Data Availability

The underlying data are included in the text.

## Supplementary Materials

Supplementary Materials Supplemental File: density plot for the variable IgE.
