# Supplementary figures and images for: Increased IgE Deposition in Appendicular Tissue Specimens Is Compatible with a Type I Hypersensitivity Reaction in Acute Appendicitis
Source: Mediators Inflamm. 2021 Oct 18;2021:4194859. doi: 10.1155/2021/4194859 (PMC8545569; doi:10.1155/2021/4194859)

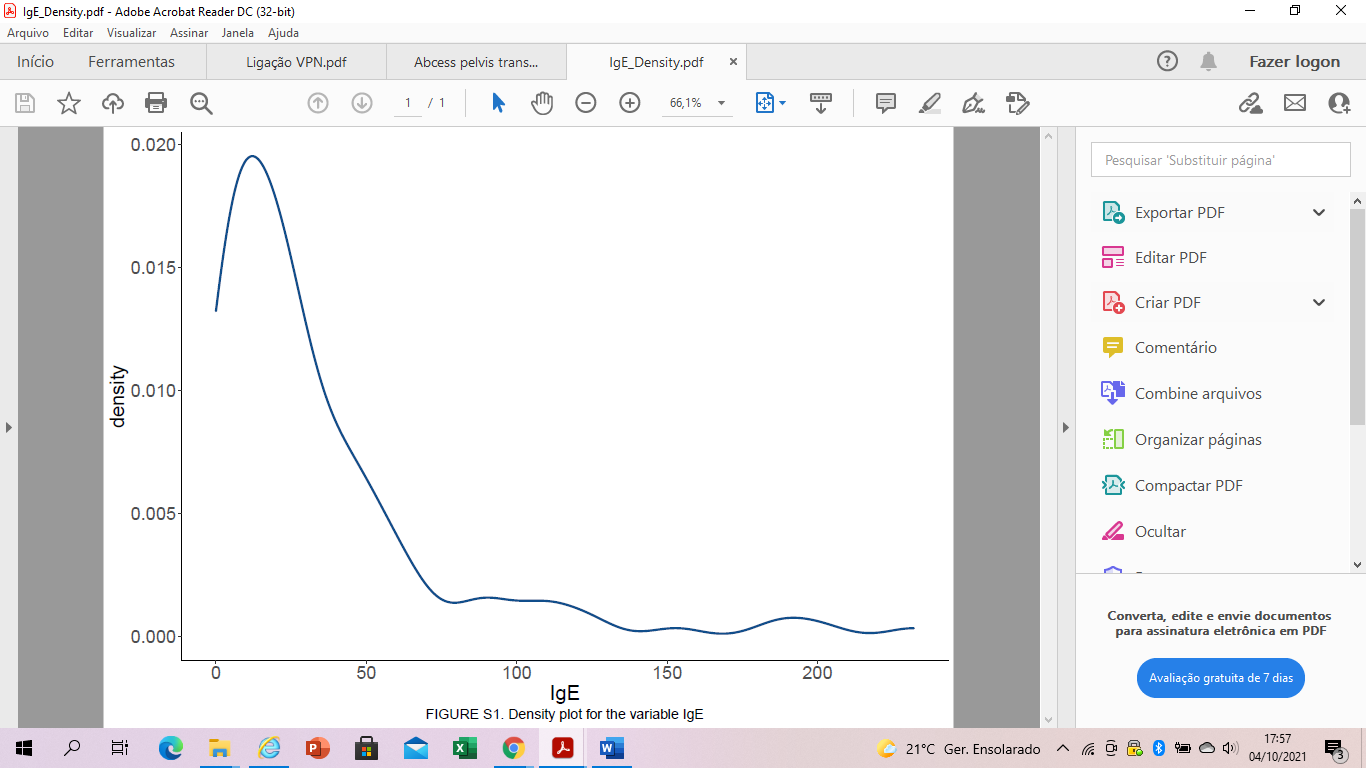


Suplemental File: Density plot for the variable IgE

Supplement: Supplementary Materials — Supplemental File: density plot for the variable IgE. [file 4194859.f1.docx]
